# Long-term follow-up of patients with relapsing multiple sclerosis from the CLARITY/CLARITY Extension cohort of CLASSIC-MS: An ambispective study

**DOI:** 10.1177/13524585231161494

**Published:** 2023-04-03

**Authors:** Gavin Giovannoni, Alexey Boyko, Jorge Correale, Gilles Edan, Mark S Freedman, Xavier Montalban, Kottil Rammohan, Dusan Stefoski, Bassem Yamout, Thomas Leist, Aida Aydemir, Laszlo Borsi, Elisabetta Verdun di Cantogno

**Affiliations:** Blizard Institute, Barts and The London School of Medicine and Dentistry, Queen Mary University of London, London, UK; Department of Neurology, Neurosurgery and Medical Genetics, Federal Center of Brain Research and Neurotechnologies, Pirogov Russian National Research Medical University, Moscow, Russia; Department of Neurology, FLENI Institute, Buenos Aires, Argentina; Department of Neurology, University Hospital of Rennes, Rennes, France; Department of Medicine and the Ottawa Hospital Research Institute, University of Ottawa, Ottawa, ON, Canada; Department of Neurology-Neuroimmunology, Centre d’Esclerosi Múltiple de Catalunya (Cemcat), Hospital Universitario Vall d’Hebron, Barcelona, Spain; MS Research Center, School of Medicine, University of Miami, Miami, FL, USA; Department of Neurological Sciences, Rush Medical College, Chicago, IL, USA; Neurology Institute, Harley Street Medical Center, Abu Dhabi, UAE/American University of Beirut Medical Center, Beirut, Lebanon; Division of Clinical Neuroimmunology, Comprehensive MS Center, Jefferson University, Philadelphia, PA, USA; EMD Serono Research & Development Institute, Inc., Billerica, MA, USA, an affiliate of Merck KGaA; Merck Healthcare KGaA, Darmstadt, Germany; EMD Serono Research & Development Institute, Inc., Billerica, MA, USA, an affiliate of Merck KGaA

**Keywords:** Cladribine tablets, CLARITY, CLARITY Extension, disability, disease-modifying therapy, employment, Expanded Disability Status Scale, multiple sclerosis

## Abstract

**Background:**

CLASSIC-MS evaluated the long-term efficacy of cladribine tablets in patients with relapsing multiple sclerosis.

**Objective:**

Report long-term mobility and disability beyond treatment courses received in CLARITY/CLARITY Extension.

**Methods:**

This analysis represents CLASSIC-MS patients who participated in CLARITY with/without participation in CLARITY Extension, and received ⩾1 course of cladribine tablets or placebo (*N* = 435). Primary objective includes evaluation of long-term mobility (no wheelchair use in the 3 months prior to first visit in CLASSIC-MS and not bedridden at any time since last parent study dose (LPSD), i.e. Expanded Disability Status Scale (EDSS) score <7). Secondary objective includes long-term disability status (no use of an ambulatory device (EDSS < 6) at any time since LPSD).

**Results:**

At CLASSIC-MS baseline, mean ± standard deviation EDSS score was 3.9 ± 2.1 and the median time since LPSD was 10.9 (range = 9.3–14.9) years. Cladribine tablets–exposed population: 90.6% (*N* = 394), including 160 patients who received a cumulative dose of 3.5 mg/kg over 2 years. Patients not using a wheelchair and not bedridden: exposed, 90.0%; unexposed, 77.8%. Patients with no use of an ambulatory device: exposed, 81.2%; unexposed, 75.6%.

**Conclusion:**

With a median 10.9 years’ follow-up after CLARITY/CLARITY Extension, findings suggest the sustained long-term mobility and disability benefits of cladribine tablets.

## Introduction

Multiple sclerosis (MS) is a chronic, inflammatory, demyelinating, and neurodegenerative disease of the central nervous system that is most commonly diagnosed in young adults between the ages of 20 and 50 years,^1–3^ and is typically characterized by frequent relapses paralleled by disability progression and cognitive impairment.^4^

Cladribine tablets (3.5 mg/kg cumulative dose over 2 years) is a high-efficacy disease-modifying therapy (DMT) approved for use in the treatment of relapsing MS, having shown significant benefits in both treatment naïve and treatment-experienced patients.^5–7^ This agent has novel posology among available DMTs, in that it comprises a short treatment course at the beginning of the first and second months of two consecutive treatment years; thereafter, no further treatment with cladribine tablets is required in years 3 and 4, in view of sustained efficacy.

The CLARITY (**CLA**d**RI**bine **T**ablets for treating MS orall**Y**) study, which recruited patients between April 2005 and January 2007, was conducted at a time when limited high-efficacy treatments were available and the diagnosis of MS was based on the older 2001 McDonald criteria. Despite this, the results from CLARITY showed that short-course treatment with cladribine tablets significantly reduced relapse rates, the risk of disability progression, and improved magnetic resonance imaging (MRI) outcomes.^5^ In turn, CLARITY Extension provided further evidence of the sustained efficacy of cladribine tablets.^6^ Subsequent analysis of CLARITY Extension has indicated the sustained benefits of cladribine tablets in terms of no evidence of disease activity (NEDA-3), and for up to 6 years from the baseline of CLARITY.^8^

The CLASSIC-MS study (NCT03961204) was designed to further explore the long-term efficacy and durability of the effect of cladribine tablets beyond the two annual treatment courses in patients enrolled in the parent trials of the Phase III development program (CLARITY, CLARITY Extension, and ORACLE MS [**ORA**l **CL**adribine in **E**arly **M**ultiple **S**clerosis]). The analysis presented here focuses on the CLASSIC-MS patient population previously enrolled in CLARITY with or without subsequent enrollment to CLARITY Extension. Findings for the ORACLE MS cohort are to be reported elsewhere.

## Methods

### Study design and endpoints

**Figure 1.**
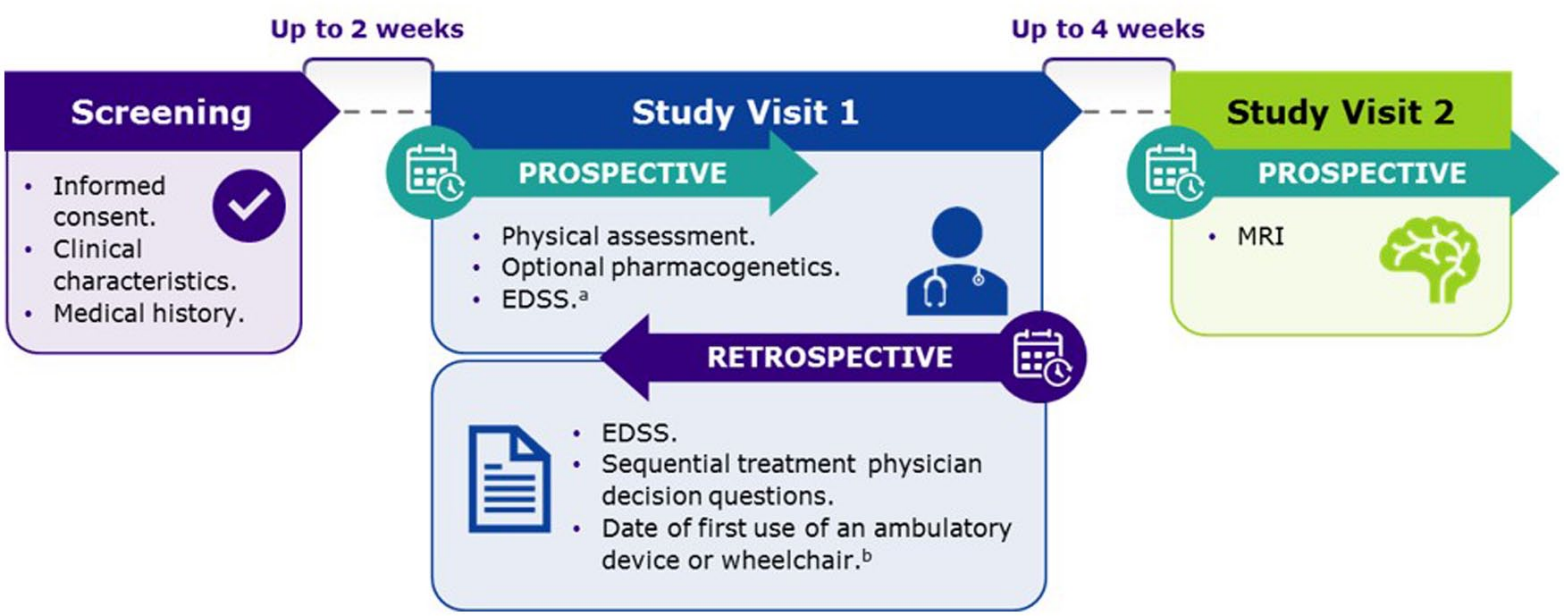
CLASSIC-MS study design. EDSS: Expanded Disability Status Scale; MRI: magnetic resonance imaging. ^a^Can also be administered by telephone instead of in-person at clinic at Study Visit 1. ^b^May be determined through retrospective chart review and/or at Study Visit 1, for example, if conversion or disability progression occurred between last regular clinical visit and Study Visit 1.

CLASSIC-MS was an exploratory, low-interventional, multicenter, ambispective, Phase IV study of patients with MS (Figure 1), in which the assessment of patients took place across 98 centers in 29 countries between 2019 and 2021.

The analysis presented here concerns data for patients who participated in CLARITY with or without subsequent enrollment to CLARITY Extension, for which the median time to follow-up in CLASSIC-MS since the last parent study dose (LPSD) was 10.9 (range = 9.3–14.9) years. The time since LPSD was defined as the time since the last treatment dose of cladribine tablets or placebo during the parent study; this timing varies between patients depending on their enrollment in the CLARITY Extension study and the number of treatment courses received during CLARITY/CLARITY Extension (Supplemental Figure 1). To be eligible for inclusion in the current analysis, patients must have received ⩾1 course of cladribine tablets or placebo during the parent studies and must have been able to provide informed consent at the time of enrollment.

During the first study visit of CLASSIC-MS (hereafter referred to as “Study Visit 1”), retrospective data on Expanded Disability Status Scale (EDSS) score,^9^ use of ambulatory device(s), relapses, and subsequent use of DMTs were collected along with employment status. For the purposes of analysis, “actively employed” included people who were “employed for wages,” “self-employed,” or considered themselves a “homemaker” at the time of Study Visit 1.

The primary objective of CLASSIC-MS was to evaluate long-term mobility by determining the proportion of patients not using a wheelchair in the 3 months prior to Study Visit 1 and not bedridden at any time since LPSD, as determined by a level of functioning consistent with an EDSS score <7. Where EDSS scores were not available, alternative clinical descriptions in the medical records were used.

Secondary objectives were to assess long-term disability status by determining the proportion of patients not using an ambulatory device since LPSD. This was determined by a level of functioning consistent with an EDSS score <6 or alternative clinical descriptions.

The tertiary objectives were to determine real-world treatment patterns by assessing the number, type, and timing of subsequent DMT use, and the durability of clinical outcomes as assessed by the time from first [F]/[L]PSD to use of an ambulatory device.

In this study, a positive treatment response during the 4-year period since LPSD was defined using three variables, with responses categorized as “Yes,” “No,” and “Not determined”:

(a) Not using further DMT(s);
(b) No evidence of disease reactivation based on medical records and investigator assessments of clinical outcomes; and
(c) Not using further DMT(s) and no evidence of disease reactivation.

Safety data were not evaluated as part of the CLASSIC-MS study, having been reported on as part of the parent studies.

### Statistical analysis

Data evaluation and interpretation are based on point estimates and 95% confidence intervals (CIs). Due to the exploratory and hypothesis-generating nature of the study, no testing of formal statistical hypotheses or adjustments for multiple comparisons was performed. Time-to-event analyses are presented using the Kaplan–Meier estimates and cumulative incidence curves. Findings are presented according to patient exposure/non-exposure to cladribine tablets in the parent studies (i.e. CLARITY/CLARITY Extension), and separately for those who received a cladribine tablets dose of 3.5 mg/kg over 2 years. Analyses were performed using SAS^®^ software version 9.4 or higher.

## Results

A total of 435 patients from CLARITY with or without subsequent enrollment to CLARITY Extension (of whom 345 patients participated in both studies) were included in this analysis of CLASSIC-MS. This population had a median age of 52.5 (range = 32–79) years and was predominantly female (67.8%). Concerning disability, patients had a median EDSS score of 3.5 (range = 0.0–9.0) at Study Visit 1 of CLASSIC-MS compared with 2.5 (range = 0.0–5.5) at the parent study baseline. For patients exposed to cladribine tablets, there was a 1.0-point increase in median EDSS scores between the parent study baseline and Study Visit 1 compared with a 1.5-point increase in patients who were never exposed to active treatment. Of the 435 patients included in this analysis, 90.6% (394/435) had been exposed to cladribine tablets in the parent studies, with 160 patients having received a cumulative dose of 3.5 mg/kg over 2 years, with the other 234 patients having been exposed to varying doses of cladribine tablets during the parent studies (Supplemental Figure 1). Baseline characteristics of the exposed and never-exposed cohorts of CLASSIC-MS patients from CLARITY/CLARITY Extension were largely similar, as shown in Table 1. Overall, baseline disease characteristics of patients enrolled on CLASSIC-MS were similar to those who were not enrolled on the study (Supplemental Table 1).

**Table 1.** Patient demographics and disease characteristics at parent study baseline and Study Visit 1 of CLASSIC-MS: CLARITY/CLARITY Extension cohort.

Parameter	Never exposed to cladribine tablets^a^ (*N* = 41)	Exposed to cladribine tablets		Total (*N* = 435)
		All exposed patients^b^ (*N* = 394)	Subgroup exposed to 3.5 mg/kg dose^c^ (*N* = 160)	
Female, *n* (%)	31 (75.6)	264 (67.0)	103 (64.4)	295 (67.8)
Age at Study Visit 1 (years), mean ± SD	51.6 ± 10.25	52.8 ± 9.56	51.7 (9.76)	52.7 ± 9.62
Disease duration at Study Visit 1^d^ (years), mean ± SD	22.38 ± 6.85	22.36 ± 6.99	21.32 ± 6.21	22.36 ± 6.97
Time since the last dose in the parent study to Study Visit 1 (years)				
Mean ± SD	13.50 ± 0.47	11.14 ± 1.17	11.05 ± 1.15	11.35 ± 1.31
Median (range)	13.40 (12.4–14.5)	10.79 (9.3–14.9)	10.65 (9.5–14.4)	10.89 (9.3–14.9)
Duration of treatment during parent study (years)^e^				
Mean ± SD	0.85 ± 0.31	1.86 ± 1.27	1.01 ± 0.05	1.77 ± 1.25
Median (range)	0.99 (0.1–1.2)	1.01 (0.0–4.6)	0.99 (0.9–1.2)	1.00 (0.0–4.6)
EDSS score at parent study baseline				
Mean ± SD	2.74 ± 1.33	2.82 ± 1.29	2.74 ± 1.31	2.82 ± 1.29
Median (range)	3.00 (0.0–5.5)	2.50 (0.0–5.5)	2.50 (0.0–5.5)	2.50 (0.0–5.5)
EDSS score at Study Visit 1				
Mean ± SD	4.50 ± 2.59	3.82 ± 2.01	3.78 ± 2.07	3.87 ± 2.07
Median (range)	4.50 (0.0–9.0)	3.50 (0.0–9.0)	3.50 (0.0–9.0)	3.50 (0.0–9.0)
Number of relapses in the 12 months before enrollment to parent study, mean ± SD	1.6 ± 0.78	1.3 ± 0.59	1.3 ± 0.62	1.3 ± 0.62
Type of MS at CLASSIC-MS screening, *n* (%)				
RRMS	29 (70.7)	292 (74.1)	116 (72.5)	321 (73.8)
SPMS	12 (29.3)	102 (25.9)	44 (27.5)	114 (26.2)
Prior use of DMT at parent study baseline, *n* (%)	11 (26.8)	83 (21.1)	34 (21.3)	94 (21.6)
HDA^f^ status at parent study baseline, *n* (%)	18 (43.9)	110 (27.9)	48 (30.0)	128 (29.4)
Employment status at Study Visit 1, *n* (%)				
Employed for wages	8 (19.5)	146 (37.1)	60 (37.5)	154 (35.4)
Self-employed	0 (0)	23 (5.8)	10 (6.3)	23 (5.3)
Homemaker	3 (7.3)	32 (8.1)	16 (10.0)	35 (8.0)
Retired	7 (17.1)	74 (18.8)	26 (16.3)	81 (18.6)
Out of work/unable to work	14 (34.1)	82 (20.8)	34 (21.3)	96 (22.1)
Unknown^g^	9 (22.0)	37 (9.4)	14 (8.8)	46 (10.6)

DMT: disease-modifying therapy; EDSS: Expanded Disability Status Scale; FPSD: first parent study dose; HDA: high disease activity; LPSD: last parent study dose; MS: multiple sclerosis; RRMS: relapsing-remitting multiple sclerosis; SD: standard deviation; SPMS: secondary progressive multiple sclerosis.

a Never-exposed cohort received only placebo during the parent studies.

b Exposed cohort includes all patients who received ⩾1 dose of cladribine tablets during the parent studies.

c A subgroup of the exposed cohort in which patients received 3.5 mg/kg cumulative dose over 2 years during the parent studies (*N* = 160/394).

d Disease duration = (Study Visit 1 − date of MS diagnosis + 1)/365.25.

e Treatment duration = (LPSD − FPSD + 1)/365.25.

f Defined as patients with ⩾2 relapses in the 12 months prior to parent study entry, regardless of prior DMT use, OR patients with ⩾1 relapse in the previous 12 months and ⩾1 T1 gadolinium-enhancing lesion or ⩾9 T2 lesions while on therapy with other DMTs.

g Includes those with missing/not reported data or information not collected at study site.

### Primary endpoint (median 10.9 years since LPSD)

**Figure 2.**
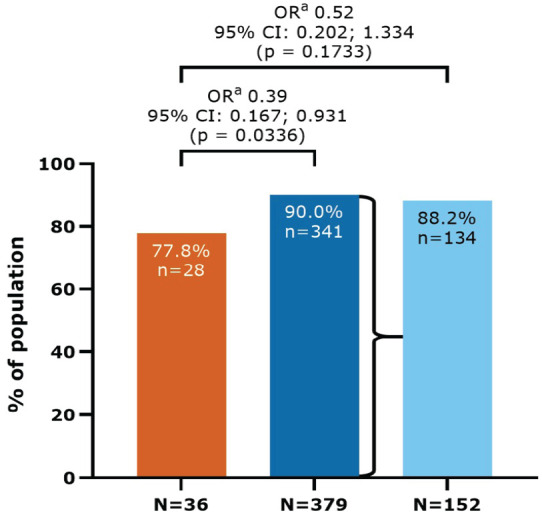
Patients not using a wheelchair in the 3 months prior to Study Visit 1 and not bedridden at any time since LPSD (EDSS < 7): CLARITY/CLARITY Extension cohort. CI: confidence interval; EDSS: Expanded Disability Status Scale; LPSD: last parent study dose; OR: odds ratio. Missing data were not included in the analysis (*n* = 5, *n* = 15, and *n* = 8 for never exposed, exposed, and exposed to cladribine tablets 3.5 mg/kg over 2 years, respectively). ^a^From a logistic regression model with fixed effects for treatment group and disease duration. ^b^Never-exposed cohort received only placebo during the parent studies. ^c^Exposed cohort includes all patients who received ⩾1 dose of cladribine tablets during the parent studies. ^d^A subgroup of the exposed cohort in which patients received 3.5 mg/kg cumulative dose over 2 years during the parent studies (*N* = 160/394).

In this study population, 88.9% of evaluable patients (369/415) were not using a wheelchair in the 3 months prior to Study Visit 1 and were not bedridden at any time since LPSD (i.e. EDSS < 7). This represented 77.8% (28/36) of patients who were never exposed to active treatment, compared with 90.0% (341/379) of patients who were exposed to cladribine tablets (odds ratio = 0.39, 95% CI = 0.17–0.93; *p* = 0.034) (Figure 2). For patients receiving cladribine tablets 3.5 mg/kg over 2 years, 88.2% (134/152) were not using a wheelchair and were not bedridden during these same time periods. When compared with the never-exposed cohort (36/41), this provided an odds ratio of 0.52 (95% CI = 0.20–1.33; *p* = 0.173).

**Figure 3.**
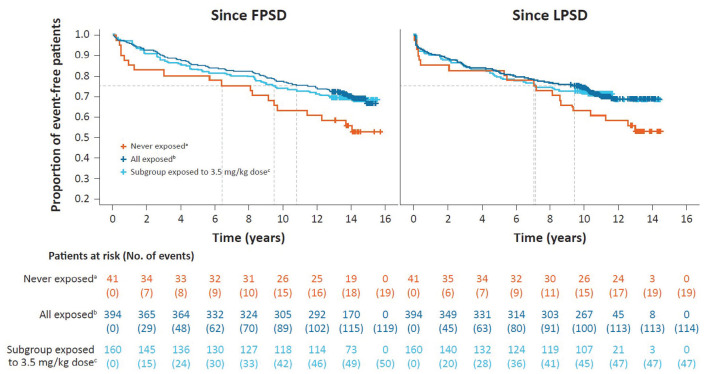
Kaplan–Meier curve for time to use of an ambulatory device since parent study dosing in CLARITY/CLARITY Extension. ^a^Never-exposed cohort received only placebo during the parent studies. ^b^Exposed cohort includes all patients who received ⩾1 dose of cladribine tablets during the parent studies. ^c^A subgroup of the exposed cohort in which patients received 3.5 mg/kg cumulative dose over 2 years during the parent studies (*N* = 160/394).

In terms of time to the first use of an ambulatory device since LPSD (tertiary endpoint), 28.9% (114/394) of patients exposed to cladribine tablets and 46.3% (19/41) of never-exposed patients had an event with an estimated time of 9.9 and 7.2 years for 25% of patients to reach an event, respectively (Figure 3).

### Secondary endpoint (median 10.9 years since LPSD)

**Figure 4.**
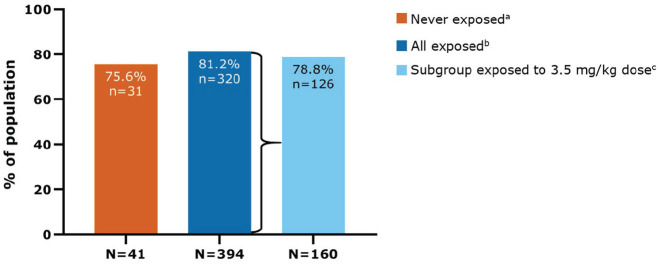
Patients who were not using an ambulatory device at any time since last parent study dose (EDSS < 6) in CLARITY/CLARITY Extension. EDSS: Expanded Disability Status Scale. ^a^Never-exposed cohort received only placebo during the parent studies. ^b^Exposed cohort includes all patients who received ⩾1 dose of cladribine tablets during the parent studies. ^c^A subgroup of the exposed cohort in which patients received 3.5 mg/kg cumulative dose over 2 years during the parent studies (*N* = 160/394).

In this study population, 80.7% (351/435) of patients did not use an ambulatory device at any time since LPSD (i.e. EDSS < 6). For patients who were never exposed to active treatment, the corresponding proportion was 75.6% (31/41) compared with 81.2% (320/394) of patients who were exposed to cladribine tablets (Figure 4). For patients receiving cladribine tablets 3.5 mg/kg over 2 years, 78.8% (126/160) did not use an ambulatory device at any time since LPSD.

### Response at 4 years since LPSD

Findings of the 4-year responder analyses indicated that 63.4% (276/435) of patients did not use a subsequent DMT; 48.0% (209/435) showed no evidence of disease reactivation, and 32.6% (142/435) did not use a subsequent DMT and also showed no evidence of disease reactivation (Table 2).

**Table 2.** Responder findings of CLASSIC-MS: CLARITY/CLARITY Extension cohort in the 4 years since LPSD.

Responder definition, *n* (%)	Never exposed to cladribine tablets^a^ (*N* = 41)	Exposed to cladribine tablets		Total (*N* = 435)
		All exposed patients^b^ (*N* = 394)	Subgroup exposed to 3.5 mg/kg dose^c^ (*N* = 160)	
A. Not using further DMTs				
Yes	15 (36.6)	261 (66.2)	106 (66.3)	276 (63.4)
No	24 (58.5)	108 (27.4)	41 (25.6)	132 (30.3)
Not determined	2 (4.9)	25 (6.3)	13 (8.1)	27 (6.2)
B. No evidence of disease reactivation				
Yes	11 (26.8)	198 (50.3)	80 (50.0)	209 (48.0)
No	28 (68.3)	178 (45.2)	70 (43.8)	206 (47.4)
Not determined	2 (4.9)	18 (4.6)	10 (6.3)	20 (4.6)
C. Not using further DMTs and no evidence of disease reactivation				
Yes	6 (14.6)	136 (34.5)	57 (35.6)	142 (32.6)
No	34 (82.9)	229 (58.1)	89 (55.6)	263 (60.5)
Not determined	1 (2.4)	29 (7.4)	14 (8.8)	30 (6.9)

DMT: disease-modifying therapy; LPSD: last parent study dose.

a Never-exposed cohort received only placebo during the parent studies.

b Exposed cohort includes all patients who received ⩾1 dose of cladribine tablets during the parent studies.

c A subgroup of the exposed cohort in which patients received 3.5 mg/kg cumulative dose over 2 years during the parent studies (*N* = 160/394).

When analyzed by cohort, 66.2% (261/394) of patients exposed to cladribine tablets used no subsequent DMT(s) compared with 36.6% (15/41) in the never-exposed cohort. No evidence of disease reactivation was observed in 50.3% (198/394) of patients exposed to cladribine tablets compared with 26.8% (11/41) in the never-exposed cohort. For patients not using a subsequent DMT *and* showing NEDA, 34.5% (136/394) of patients exposed to cladribine tablets met these criteria compared with 14.6% (6/41) of patients in the never-exposed cohort. For patients receiving cladribine tablets 3.5 mg/kg over 2 years, results for the 4-year responder analyses were comparable to the exposed cohort. Results also indicate that patients with high relapse activity responded well to treatment with cladribine tablets (Supplemental Table 2).

### Subsequent DMT use (median 10.9 years since LPSD)

Over the period since LPSD, 53.1% (231/435) of patients did not use any subsequent DMTs. The majority of patients who used a subsequent treatment received a platform injectable (137/204, 67.2%), namely, interferons (94/137, 68.6%) (Supplemental Table 3). These subsequent DMTs are reflective of those available in the intervening period (2010–2021) after the completion of the parent studies.

**Figure 5.**
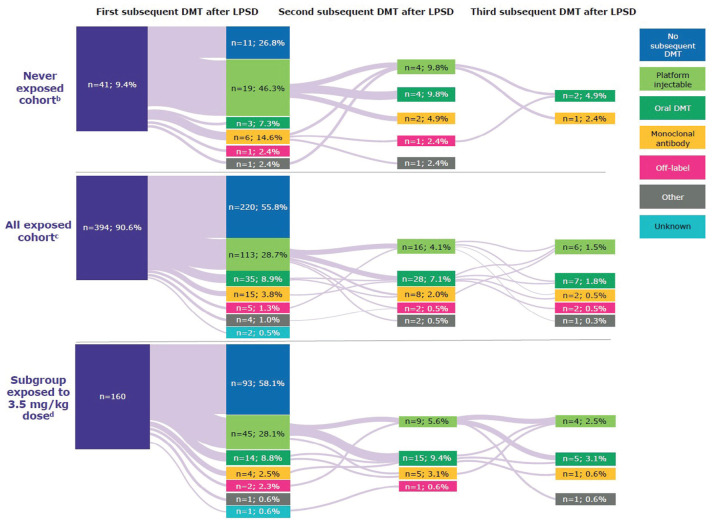
Patterns of DMT use at any time since last parent study dose in CLARITY/CLARITY Extension, by exposure to cladribine tablets. DMT: disease-modifying therapy. ^a^Subsequent DMTs are reflective of those available in the intervening period (2010–2021) after completion of the parent studies. ^b^Never-exposed cohort received only placebo during the parent studies. ^c^Exposed cohort includes all patients who received ⩾1 dose of cladribine tablets during the parent studies. ^d^A subgroup of the exposed cohort in which patients received 3.5 mg/kg cumulative dose over 2 years during the parent studies (*N* = 160/394).

Patients exposed to cladribine tablets during the parent studies were less likely to use further DMTs after LPSD. This is indicated by 55.8% (220/394) of the exposed cohort, versus 26.8% (11/41) in the never-exposed cohort, receiving no subsequent treatments during follow-up (Figure 5). For patients receiving cladribine tablets 3.5 mg/kg over 2 years, 58.1% (93/160) received no further DMTs after LPSD.

**Figure 6.**
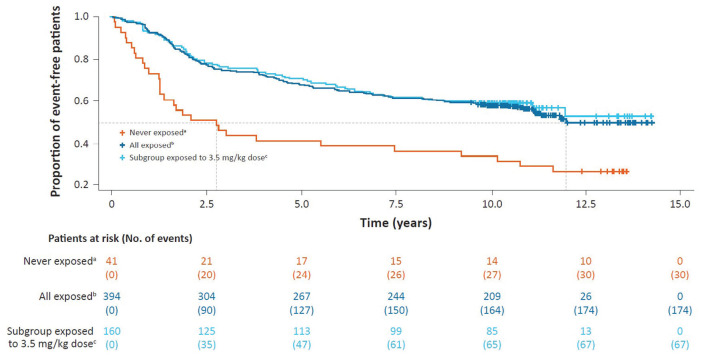
Kaplan–Meier curve for time to first subsequent DMT after last parent study dose in CLARITY/CLARITY Extension. DMT: disease-modifying therapy. ^a^Never-exposed cohort received only placebo during the parent studies. ^b^Exposed cohort includes all patients who received ⩾1 dose of cladribine tablets during the parent studies. ^c^A subgroup of the exposed cohort in which patients received 3.5 mg/kg cumulative dose over 2 years during the parent studies (*N* = 160/394).

In terms of time-to-event analysis, patients exposed to cladribine tablets had an estimated median time of 12.0 years until the first subsequent DMT; the corresponding timeframe for patients never exposed to cladribine tablets was 2.8 years (Figure 6). The corresponding time-to-event analysis for the subgroup receiving 3.5 mg/kg indicates that the data are similar to those for the exposed cohort (Figure 6).

A low proportion of patients received a second subsequent DMT following treatment with cladribine tablets; 14.2% (56/394) of patients exposed to cladribine tablets and 29.2% (12/41) of never-exposed patients. For patients receiving cladribine tablets 3.5 mg/kg over 2 years, 18.8% (30/160) received a second subsequent DMT.

The proportions of patients receiving a third subsequent DMT were lower still; 7.3% (3/41) never exposed, 4.6% (18/394) exposed to cladribine tablets, and 6.9% (11/160) of those who received the 3.5 mg/kg dose over 2 years.

### Relapses (median 10.9 years since LPSD)

During the time period since LPSD to Study Visit 1, a total of 200 patients did not experience a relapse. The proportion of patients in the exposed cohort who were relapse-free was approximately two times higher than that observed in the cohort of never-exposed patients: 48.0% (189/394) and 26.8% (11/41), respectively (Table 3). The annualized relapse rate (ARR) since LPSD for patients exposed to cladribine tablets was 0.12 (95% CI = 0.11–0.14), approximately half the ARR of the never-exposed cohort (0.23 (95% CI = 0.19–0.27)). For patients receiving cladribine tablets 3.5 mg/kg over 2 years, the ARR during the time period since LPSD was 0.13 (95% CI = 0.11–0.14). Similar trends were apparent for the analysis of relapse rates since first parent study dose (FPSD) (Table 3).

**Table 3.** Number of relapses since parent study dosing to Study Visit 1 of CLASSIC-MS: CLARITY/CLARITY Extension cohort.

	Never exposed to cladribine tablets^a^ (*N* = 41)	Exposed to cladribine tablets		Total (*N* = 435)
		All exposed patients^b^ (*N* = 394)	Subgroup exposed to 3.5 mg/kg dose^c^ (*N* = 160)	
Annualized relapse rate, *n* (95% CI)^d^				
Since FPSD	0.26 (0.22–0.31)	0.16 (0.15–0.17)	0.17 (0.16–0.19)	0.17 (0.16–0.18)
Since LPSD	0.23 (0.19–0.27)	0.12 (0.11–0.14)	0.13 (0.11–0.14)	0.14 (0.13–0.15)
Number of relapses since FPSD, *n* (%)				
0	7 (17.1)	124 (31.5)	39 (24.4)	131 (30.1)
1	7 (17.1)	79 (20.1)	34 (21.3)	86 (19.8)
2	7 (17.1)	65 (16.5)	29 (18.1)	72 (16.6)
3	7 (17.1)	39 (9.9)	21 (13.1)	46 (10.6)
4	1 (2.4)	27 (6.9)	9 (5.6)	28 (6.4)
5	1 (2.4)	19 (4.8)	10 (6.3)	20 (4.6)
⩾6	11 (26.8)	41 (10.4)	18 (11.3)	52 (12.0)
Number of relapses since LPSD, *n* (%)				
0	11 (26.8)	189 (48.0)	75 (46.9)	200 (46.0)
1	7 (17.1)	86 (21.8)	33 (20.6)	93 (21.4)
2	6 (14.6)	50 (12.7)	27 (16.9)	56 (12.9)
3	5 (12.2)	27 (6.9)	7 (4.4)	32 (7.4)
4	3 (7.3)	13 (3.3)	5 (3.1)	16 (3.7)
5	1 (2.4)	7 (1.8)	4 (2.5)	8 (1.8)
⩾6	8 (19.5)	22 (5.6)	9 (5.6)	30 (69.0)

CI: confidence interval; FPSD: first parent study dose; LPSD: last parent study dose.

a Never-exposed cohort received only placebo during the parent studies.

b Exposed cohort includes all patients who received ⩾1 dose of cladribine tablets during the parent studies.

c A subgroup of the exposed cohort in which patients received 3.5 mg/kg cumulative dose over 2 years during the parent studies (*N* = 160/394).

d Annualized relapse rate calculated as the (total number of relapses × 365.25)/total time on study until Study Visit 1. Confidence intervals were estimated using a Poisson regression model of the relapse count as dependent variable, fixed effect for treatment group, and the log of time on study as offset variable.

### Employment (median 10.9 years since LPSD)

Of the 435 patients included in this analysis, 48.7% (212/435) were in employment at Study Visit 1 (Table 1). The proportion of patients in active employment at Study Visit 1 was higher in the exposed cohort compared to the never-exposed cohort; 51.0% (201/394) and 27.5% (11/40), respectively.

## Discussion

Early treatment initiation is critical to the optimization of outcomes in people living with MS. Indeed, evidence-based clinical practice guidelines for the management of these individuals support prompt treatment decisions such as the use of high-efficacy DMTs earlier in the disease course, for appropriate patients.^10^ Such treatment decisions incorporate the degree of disease activity and other patient, clinical, biomarker, and intangible (e.g. reimbursement) factors, a key aim being the ultimate prevention of disability accumulation. However, the majority of high-efficacy DMTs achieve this benefit by applying continuous immunosuppression, which may have a cumulative safety risk for patients. The overall findings of the present analysis also raise an interesting question as to the effects of timing of initiation of high-efficacy DMTs and long-term outcomes. Data from the MSBase and Swedish MS registries, for example, have identified that early initiation of high-efficacy therapies (within 2 years of disease onset) had a beneficial effect on disability when compared with later treatment initiation.^11^ The exploratory, ambispective CLASSIC-MS study, with a median of 10.9 years’ follow-up since LPSD, therefore provides important new information on the long-term efficacy of cladribine tablets for patients who originally participated in CLARITY with or without subsequent enrollment in CLARITY Extension.

The baseline median EDSS score of the CLARITY/CLARITY Extension population enrolled to CLASSIC-MS was 2.50, and this remained relatively stable over the median follow-up of 10.9 years. When the results of CLASSIC-MS are broken down by treatment cohort, we observed that patients exposed to cladribine tablets had a 1.0-point increase in median EDSS score over this timeframe (including those patients receiving the 3.5 mg/kg dose); however, patients who were never exposed to active treatment had a 1.5-point increase in median scores, thus indicating a greater extent of disease worsening during follow-up. On one hand, these results are in line with those seen in long-term follow-up studies of other DMTs. Results from the Tysabri Observational Program, for example, showed that EDSS scores remained stable over a 10-year period in patients treated with natalizumab.^12^ Similarly, EDSS scores for patients treated with fingolimod remained stable over 10 years.^13^ It is important to consider that both natalizumab and fingolimod are maintenance therapies that rely on constant immunosuppression to maintain efficacy. In contrast, patients who received cladribine tablets had exposure to the therapy for only very short periods, with lymphocyte recovery that begins soon after each treatment course in Years 1 and 2.^14^

In the CLASSIC-MS study, other disability outcomes were consistent with EDSS scores for the respective exposed and never-exposed cohorts, and the subgroup of patients exposed to the cumulative 3.5 mg/kg dose. Specifically, we observed that patients who were never exposed to active treatment had seemingly worse disability outcomes compared with patients who received cladribine tablets. These are important findings since, as an example, the need to use an ambulatory device can have a detrimental impact on a person’s quality of life.^15^

Increasing EDSS scores may also impact the ability of a person with MS to remain in employment.^16^ Indeed, employment—and the known importance to personal identity—is very relevant to people living with MS, the majority of whom are diagnosed during their employment years. In the absence of cognitive, social, and emotional data in this study, the findings for employment status, therefore, represent an important proxy endpoint. It is therefore a notable finding that, at Study Visit 1, 51% of patients exposed to cladribine tablets were in employment compared to only 27.5% of never-exposed patients. While such results are covered by a caveat due to unknown employment status at the parent study baseline, findings for the never-exposed cohort are in line with reports of high rates of unemployment^17^ and early retirement^18^ among the MS community.

Following treatment in CLARITY/CLARITY Extension, we observed that patients exposed to cladribine tablets were less likely to use a subsequent DMT since LPSD, with an approximate 10% increase in the use of subsequent DMTs between the 4-year and median 10.9-year analyses (a change from 66.2% to 55.8% for patients exposed to cladribine tablets vs a change of 36.6%–26.8% in the never-exposed cohort). Nearly one-third of the patients (34.5%) exposed to cladribine did not use another DMT and had no evidence of disease reactivation 4 years after LPSD as opposed to 14.6% of never-exposed patients. This mirrors the disability and ARR findings, in that the proportion of patients not using a wheelchair in the 3 months prior to Study Visit 1 and not bedridden at any time since LPSD was 90.0% and 77.8%, respectively, for the exposed and never-exposed cohorts, while ARR in the latter cohort was almost double that observed in the exposed cohort. This greater relapse rate may have had an impact on the initial time to treatment switch findings.

### Study limitations

Due to the exploratory nature of the CLASSIC-MS study, no formal sample size calculations were conducted. The study planned to enroll 788 patients, yet a final population of 662 patients was actually included. Feasibility assessments were conducted, and there were various reasons why some of the original parent study sites were not included in CLASSIC-MS, including the absence of the former/a new site investigator, a limited number/no patients at a site, and no retrospective data on file. Although it is unknown what happened to patients who did not enroll to the CLASSIC-MS study, baseline disease characteristics of patients who did enroll were considered to be generally representative of patients from the parent studies and therefore partially addresses the potential selection bias (Supplemental Table 1). The population of CLASSIC-MS is also considered representative of the general MS population due to similarities with the recently reported mean age of MS diagnosis (32 years) and the higher proportion of female patients living with MS.^19^

The MRI data collected during the CLASSIC-MS study were limited, and therefore, it was not possible to calculate responder rates for definitions based on imaging findings. Similarly, employment status at parent study baseline was not collected, thus limiting the interpretation of employment-related results.

An important consideration, in terms of contextualizing the study findings, is that we do not know why more patients appeared to have disease reactivation than had another DMT (and the reason for switching), while the subsequent DMTs used by patients after the completion of the parent studies were reflective of those available in the intervening follow-up period (2010–2021). Indeed, there were limited high-efficacy treatments available for MS at the time of the CLARITY study (initiated in 2007). In addition, when considering the time to first subsequent DMT, it is important to consider that local access to healthcare and/or the availability of DMTs within individual countries may be a factor.

## Conclusion

The results from this analysis of the CLARITY/CLARITY Extension cohort of the CLASSIC-MS study indicate that patients treated with cladribine tablets had a lower risk of reaching EDSS 6 or 7 during the median 10.9 years of follow-up compared with patients who were never exposed to active treatment with cladribine tablets. The majority of patients who were exposed to cladribine tablets were also less likely to use further DMTs during the median 10.9-year period since LPSD. In addition, the time-to-event analyses indicated that these patients had a longer estimated median time until the first subsequent DMT (12 years vs 2.5 years for the never-exposed cohort), with better outcomes over the 4 years since LPSD in the responder analyses. Favorable outcomes were also observed in the subgroup of patients exposed to a cladribine tablets dose of 3.5 mg/kg over 2 years. Together, these findings support previous studies that have reported on the sustained efficacy of cladribine tablets following treatment.

## Supplemental Material

sj-docx-1-msj-10.1177_13524585231161494 – Supplemental material for Long-term follow-up of patients with relapsing multiple sclerosis from the CLARITY/CLARITY Extension cohort of CLASSIC-MS: An ambispective study Click here for additional data file. Supplemental material, sj-docx-1-msj-10.1177_13524585231161494 for Long-term follow-up of patients with relapsing multiple sclerosis from the CLARITY/CLARITY Extension cohort of CLASSIC-MS: An ambispective study by Gavin Giovannoni, Alexey Boyko, Jorge Correale, Gilles Edan, Mark S Freedman, Xavier Montalban, Kottil Rammohan, Dusan Stefoski, Bassem Yamout, Thomas Leist, Aida Aydemir, Laszlo Borsi and Elisabetta Verdun di Cantogno in Multiple Sclerosis Journal

sj-docx-2-msj-10.1177_13524585231161494 – Supplemental material for Long-term follow-up of patients with relapsing multiple sclerosis from the CLARITY/CLARITY Extension cohort of CLASSIC-MS: An ambispective study Click here for additional data file. Supplemental material, sj-docx-2-msj-10.1177_13524585231161494 for Long-term follow-up of patients with relapsing multiple sclerosis from the CLARITY/CLARITY Extension cohort of CLASSIC-MS: An ambispective study by Gavin Giovannoni, Alexey Boyko, Jorge Correale, Gilles Edan, Mark S Freedman, Xavier Montalban, Kottil Rammohan, Dusan Stefoski, Bassem Yamout, Thomas Leist, Aida Aydemir, Laszlo Borsi and Elisabetta Verdun di Cantogno in Multiple Sclerosis Journal

sj-docx-3-msj-10.1177_13524585231161494 – Supplemental material for Long-term follow-up of patients with relapsing multiple sclerosis from the CLARITY/CLARITY Extension cohort of CLASSIC-MS: An ambispective study Click here for additional data file. Supplemental material, sj-docx-3-msj-10.1177_13524585231161494 for Long-term follow-up of patients with relapsing multiple sclerosis from the CLARITY/CLARITY Extension cohort of CLASSIC-MS: An ambispective study by Gavin Giovannoni, Alexey Boyko, Jorge Correale, Gilles Edan, Mark S Freedman, Xavier Montalban, Kottil Rammohan, Dusan Stefoski, Bassem Yamout, Thomas Leist, Aida Aydemir, Laszlo Borsi and Elisabetta Verdun di Cantogno in Multiple Sclerosis Journal
