# Supplementary material for: Long-term follow-up of patients with relapsing multiple sclerosis from the CLARITY/CLARITY Extension cohort of CLASSIC-MS: An ambispective study
Source: Mult Scler. 2023 Apr 3;29(6):719–30. doi: 10.1177/13524585231161494 (PMC10176755; doi:10.1177/13524585231161494)
Supplement: sj-docx-1-msj-10.1177_13524585231161494 – Supplemental material for Long-term follow-up of patients with relapsing multiple sclerosis from the CLARITY/CLARITY Extension cohort of CLASSIC-MS: An ambispective study [file sj-docx-1-msj-10.1177_13524585231161494.docx]

# Supplementary Appendix 1

**Supplementary Table 1.** Characteristics of CLASSIC-MS patients from CLARITY/CLARITY Extension compared with non-CLASSIC-MS patients from the parent studies (CLARITY, CLARITY Extension, and ORACLE MS)

|  | **CLASSIC-MS patients from CLARITY/CLARITY Extension (N=435)** | **All CLASSIC-MS patients**  **(N=622)** | **Non-CLASSIC-MS patients^a^ (N=1232)** |
| --- | --- | --- | --- |
| Age at parent study baseline, years (mean ± SD) | 38.5 ± 9.66 | 36.4 ± 9.66 | 37.5 ± 10.25 |
| Female, n (%) | 295 (67.8) | 444 (67.1) | 815 (66.2) |
| EDSS score at parent study baseline (mean ± SD) | 2.82 ± 1.29 | 2.43 ± 1.27 | 2.56 ± 1.38 |
| Number of relapses in the 12 months before enrollment to parent study (mean ± SD)^b^ | 1.3 ± 0.62 | 1.3 ± 0.62 | 1.4 ± 0.6 |
| Prior use of DMT at parent study baseline, n (%)^b^ | 94 (21.6) | 94 (21.6) | 293 (33.8) |
| HDA^c^ status at parent study baseline, n (%)^b^ | 128 (29.4) | 128 (29.4) | 303 (34.9) |

^a^Population includes all parent study patients (enrolled in CLASSIC-MS or not) randomized in CLARITY or ORACLE MS and who received ≥1 course of cladribine tablets or placebo. ^b^Data for ORACLE MS patients not included (227 CLASSIC-MS patients and 365 non-CLASSIC-MS patients). ^c^HDA defined as patients with ≥2 relapses in the 12 months prior to parent study entry, regardless of prior DMT use, OR patients with ≥1 relapse in the previous 12 months and ≥1 T1 gadolinium enhancing lesion or ≥9 T2 lesions while on therapy with other DMTs.

DMT, disease-modifying therapy; EDSS, Expanded Disability Status Scale; HDA, high disease activity; SD, standard deviation.

**Supplementary Table 2.** Responder findings of CLASSIC-MS: CLARITY/CLARITY Extension cohort in the 4 years since LPSD by A) high disease activity and B) non-high disease activity

| A) HDA participants | | | |
| --- | --- | --- | --- |
| **Responder definition, n (%)** | **Never exposed to cladribine tablets^a^**  **(N=18)** | **All exposed patients^b^**  **(N=110)** | **Total**  **(N=128)** |
| **Not using further DMTs** | | | |
| Yes | 3 (16.7) | 80 (72.7) | 83 (64.8) |
| No | 14 (77.8) | 21 (19.1) | 35 (27.3) |
| Not determined | 1 (5.6) | 9 (8.2) | 10 (7.8) |
| **No evidence of disease reactivation** | | | |
| Yes | 4 (22.2) | 59 (53.6) | 63 (49.2) |
| No | 13 (72.2) | 46 (41.8) | 59 (46.1) |
| Not determined | 1 (5.6) | 5 (4.5) | 6 (4.7) |
|  |  |  |  |
| B) Non-HDA participants | | | |
| **Responder definition, n (%)** | **Never exposed to cladribine tablets^a^**  **(N=23)** | **All exposed patients^b^**  **(N=284)** | **Total**  **(N=307)** |
| **Not using further DMTs** | | | |
| Yes | 12 (52.2) | 181 (63.7) | 193 (62.9) |
| No | 10 (43.5) | 87 (30.6) | 97 (31.6) |
| Not determined | 1 (4.3) | 16 (5.6) | 17 (5.5) |
| **No evidence of disease reactivation** | | | |
| Yes | 7 (30.4) | 139 (48.9) | 146 (47.6) |
| No | 15 (65.2) | 132 (46.5) | 147 (47.9) |
| Not determined | 1 (4.3) | 13 (4.6) | 14 (4.6) |

^a^Never-exposed cohort received only placebo during the parent studies. ^b^Exposed cohort includes all patients who received ≥1 dose of cladribine tablets during the parent studies.
DMT, disease-modifying therapy; HDA, high disease activity; LPSD, last parent study dose.

**Supplementary Table 3.** CLASSIC-MS patients treated with subsequent DMTs at any time since last parent study dose: CLARITY/CLARITY Extension cohort

| **Parameter, n (%)** | **Never exposed to cladribine tablets^a^**  **(N=41)** | **Exposed to cladribine tablets** | | **Total**  **(N=435)** |
| --- | --- | --- | --- | --- |
|  |  | **All exposed patients^b^**  **(N=394)** | **Subgroup exposed to 3.5 mg/kg dose^c^**  **(N=160)** |  |
| **Patients with any subsequent DMT since LPSD^d^** | **30 (73.2)** | **174 (44.2)** | **67 (41.9)** | **204 (46.9)** |
| Patients with one subsequent DMT | 18 (43.9) | 118 (29.9) | 37 (23.1) | 136 (31.3) |
| Patients with two subsequent DMTs | 9 (22.0) | 38 (9.6) | 19 (11.9) | 47 (10.8) |
| Patients with three subsequent DMTs | 3 (7.3) | 18 (4.6) | 11 (6.9) | 21 (4.8) |
| **Type of subsequent DMT received after LPSD** | | | | |
| Platform injectable therapy | 21 (51.2) | 116 (29.4) | 47 (29.4) | 137 (31.5) |
| Interferons | 17 (41.5) | 77 (19.5) | 34 (21.3) | 94 (21.6) |
| Other immunostimulants | 6 (14.6) | 54 (13.7) | 22 (13.8) | 60 (13.8) |
| Antivirals | 5 (12.2) | 21 (5.3) | 10 (6.3) | 26 (6.0) |
| Oral DMTs | 9 (22.0) | 64 (16.2) | 31 (19.4) | 73 (16.8) |
| Selective immunosuppressants | 8 (19.5) | 50 (12.7) | 25 (15.6) | 58 (13.3) |
| Other immunosuppressants | 2 (4.9) | 19 (4.8) | 8 (5.0) | 21 (4.8) |
| Purine analogues | 0 (0) | 2 (0.5) | 2 (1.3) | 2 (0.5) |
| Monoclonal antibody DMT | 9 (22.0) | 26 (6.6) | 11 (6.9) | 35 (8.0) |
| Selective immunosuppressants | 9 (22.0) | 24 (6.1) | 10 (6.3) | 33 (7.6) |
| Interleukin inhibitors | 0 (0) | 1 (0.3) | 1 (0.6) | 1 (0.2) |
| Monoclonal antibodies | 1 (2.4) | 0 (0) | 0 (0) | 1 (0.2) |
| Tumor necrosis factor alpha inhibitors | 0 (0) | 1 (0.3) | 0 (0) | 1 (0.2) |

**Supplementary Table 3.** Contd

| **Parameter, n (%)** | **Never exposed to cladribine tablets^a^**  **(N=41)** | **Exposed to cladribine tablets** | | | **Total**  **(N=435)** |
| --- | --- | --- | --- | --- | --- |
|  |  | **All exposed patients^b^**  **(N=394)** | **Subgroup exposed to 3.5 mg/kg dose^c^**  **(N=160)** | |  |
| Off-label treatment | 2 (4.9) | 9 (2.3) | 3 (1.9) | 11 (2.5) | |
| Other immunosuppressants | 0 (0) | 8 (2.0) | 2 (1.3) | 8 (1.8) | |
| Nitrogen mustard analogues | 2 (4.9) | 2 (0.5) | 1 (0.6) | 4 (0.9) | |
| Other nervous system drugs | 0 (0) | 2 (0.5) | 0 (0) | 2 (0.5) | |
| Folic acid analogues | 0 (0) | 1 (0.3) | 0 (0) | 1 (0.2) | |
| Other gynecologicals | 0 (0) | 1 (0.3) | 0 (0) | 1 (0.2) | |
| Selective immunosuppressants | 1 (2.4) | 0 (0) | 0 (0) | 1 (0.2) | |
| Other subsequent treatment | 3 (7.3) | 7 (1.8) | 2 (1.3) | 10 (2.3) | |
| Anthracyclines and related substances | 3 (7.3) | 7 (1.8) | 2 (1.3) | 10 (2.3) | |
| Unknown | 0 (0) | 2 (0.5) | 1 (0.6) | 2 (0.5) | |

^a^Never-exposed cohort received only placebo during the parent studies. ^b^Exposed cohort includes all patients who received ≥1 dose of cladribine tablets during the parent studies. ^c^A subgroup of the exposed cohort in which patients received 3.5 mg/kg cumulative dose over 2 years during the parent studies (N=160/394). ^d^Subsequent DMTs are reflective of those available at the time of the study (2010–2019). The same DMT can have multiple ATC codes, as determined by WHO-DD Version September 2020.

ATC, Anatomical Therapeutic Chemical; DMT, disease-modifying therapy; LPSD, last parent study dose; WHO-DD, World Health Organization Drug Dictionary.

**Supplementary Figure 1**. Planned treatment course for patients enrolling in CLARITY with or without subsequent enrollment to CLARITY Extension.


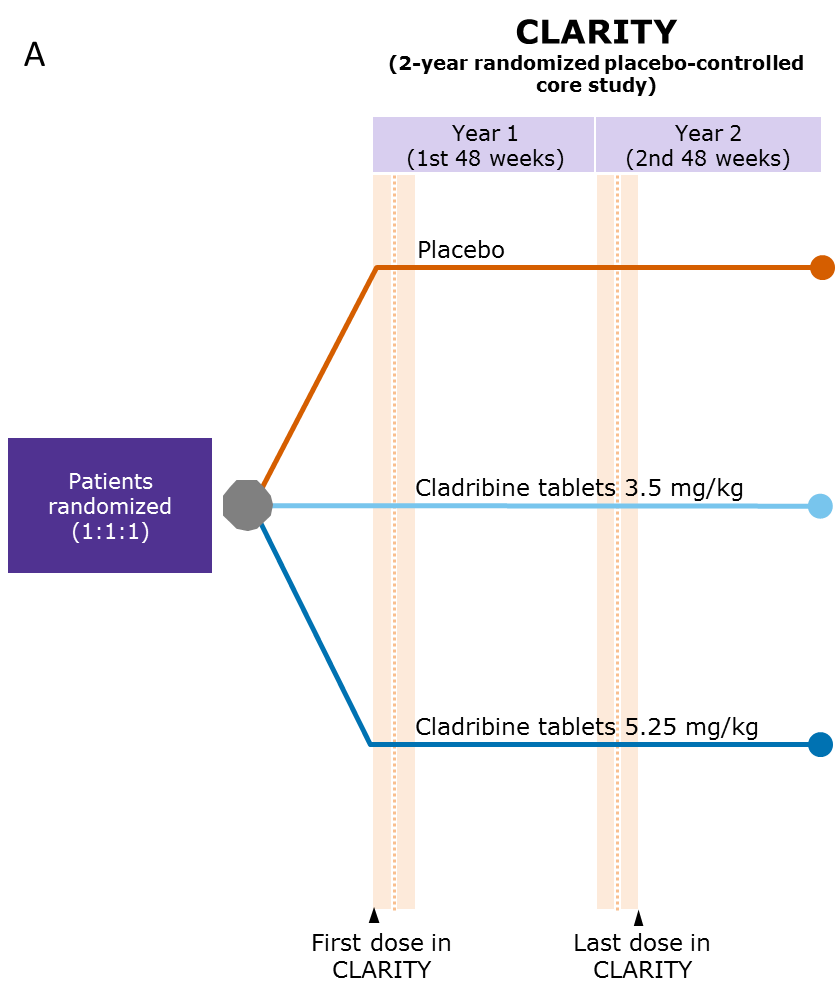


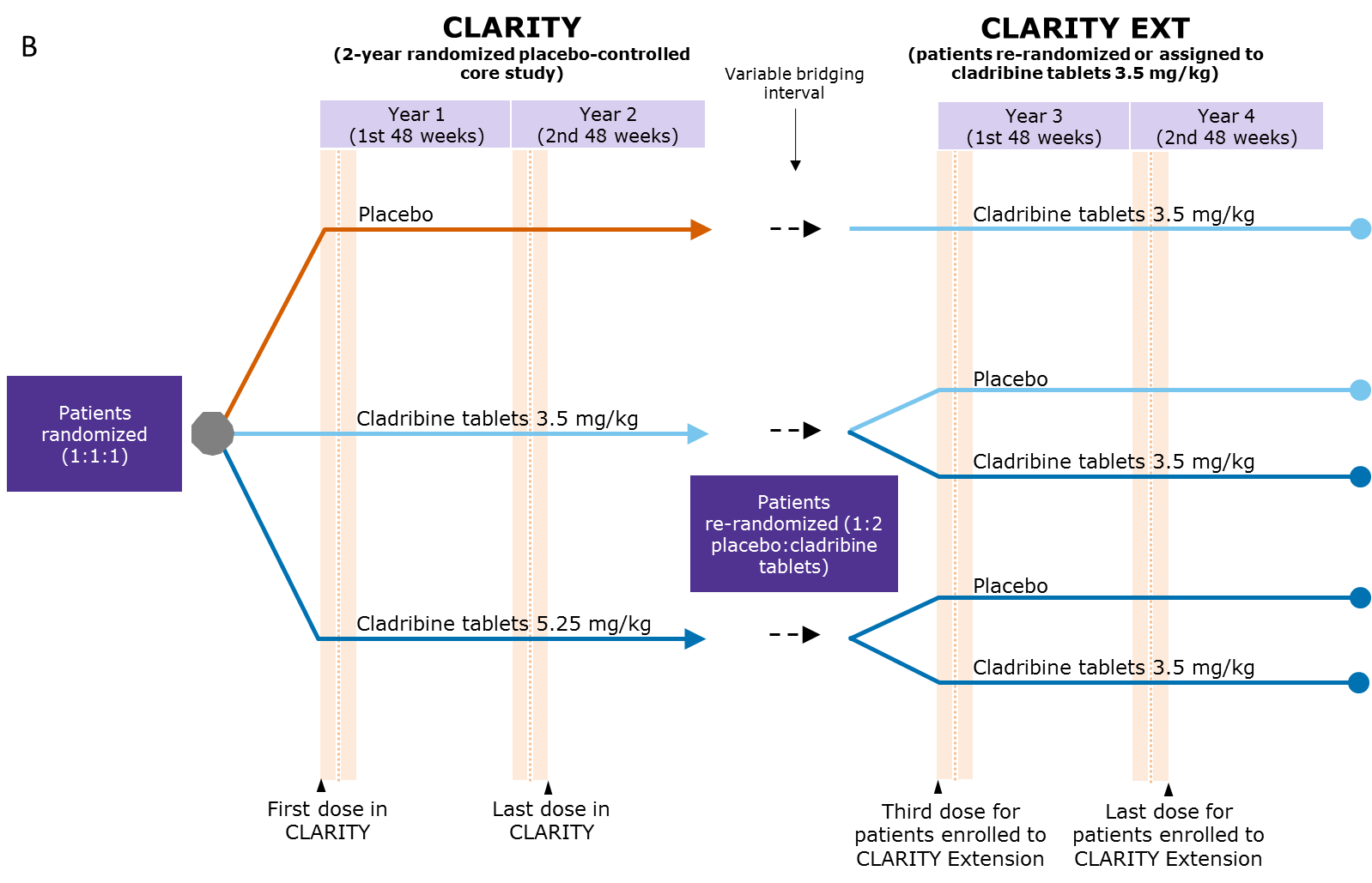


Patients were originally enrolled to the 2-year CLARITY study (A) and after completion of the core study were invited to enroll into CLARITY Extension (B). Shaded vertical bands denote the annual treatment courses in the respective parent studies. These treatment courses consisted of two treatment weeks, one at the beginning of the first month and one at the beginning of the second month of the respective treatment years. The colored treatment arms denote the cohorts in CLASSIC-MS: orange = never exposed to cladribine tablets; light blue = exposed to a cladribine tablets cumulative dose of 3.5 mg/kg in either CLARITY or CLARITY Extension; dark blue = exposed to a cladribine tablets cumulative dose of 5.25 mg/kg or more.
