# Supplementary material for: Long-term follow-up of patients with relapsing multiple sclerosis from the CLARITY/CLARITY Extension cohort of CLASSIC-MS: An ambispective study
Source: Mult Scler. 2023 Apr 3;29(6):719–30. doi: 10.1177/13524585231161494 (PMC10176755; doi:10.1177/13524585231161494)
Supplement: sj-docx-2-msj-10.1177_13524585231161494 – Supplemental material for Long-term follow-up of patients with relapsing multiple sclerosis from the CLARITY/CLARITY Extension cohort of CLASSIC-MS: An ambispective study [file sj-docx-2-msj-10.1177_13524585231161494.docx]

# Supplementary Appendix 2

Full list of ethics approvals obtained for the CLASSIC-MS study.

| **Authority Name** | **Authority Address** | **Date of Approval / Notification** | **Ethics Reference Number** |
| --- | --- | --- | --- |
| University of Sydney (RGO) | Sydney/Australia | 26/Jan/2020 | 2019/ETH12107 |
| University of Sydney Human Research Ethics Committee (HREC) | Sydney/Australia | 22/Nov/2019 | 2019/496 |
| Ethikkommission der Krankenanstalten der Barmherzigen Schwestern und der Barmherzigen Brüder | Linz/Austria | 22/Nov/2019 | EKB 04-19 |
| Ethikkommission des Landes Oberösterreich | Linz/Austria | 28/Oct/2019 | 1149/2019 |
| CHU de Liege | Liege/Belgium | 04/Sep/2019 | 2019/178 |
| Ethics Committee for Multicenter Trials | Sofia/Bulgaria | 05/Sep/2019 | ECCI-2296/31May2019 |
| Ottawa Hospital Research Ethics Board | Ottawa/Canada | 10/Dec/2019 | 20190523-01H |
| Fraser Health Research Ethics Board | Surrey/Canada | 28/Jul/2020 | 2019-083 |
| Agency for Medicinal Product and Medical Devices of Croatia, Central Ethics Committee | Zagreb/Croatia | 19/Sep/2019 | 381-15/60-19-06 |
| Eticka komise FN a LF UP Olomouc | Olomouc/Czech Republic | 13/May/2019 | 46/19 |
| Eticka komise Fakultni nemocnice Ostrava | Ostrava/Czech Republic | 30/May/2019 | 507/2019 |
| Eticka komise Vseobecne fakultni nemocnice v Praze | Praha 2/Czech Republic | 23/May/2019 | 840/19 S |
| Eticka komise IKEM a FTNsP | Praha 4 - Krc/Czech Republic | 12/Jun/2019 | 14150/19 |
| Eticka komise pro multicentricke klinicke hodnoceni Fakultni nemocnice v Motole | Praha 5/Czech Republic | 22/May/2019 | EK-543/19 |
| Eticka komise Krajska zdravotni a.s. - Nemocnice Teplice o.z. | Teplice/Czech Republic | 11/Jun/2019 | VI/19/1 |
| Research Ethics Committee of the University of Tartu | Tartu/Estonia | 30/Sep/2019 | 294/T-3 |
| Varsinais-Suomen sairaanhoitopiiri | Turku/Finland | 17/Sep/2019 | 55/1802/2019 |
| Agence Nationale de Sécurité du Médicament et des Produits de Santé | Tours cedex 9/France | 04/Jun/2019 | 2019T1-11 (2019-000069-19) (CLASSIC MS |
| Comité de Protection des Personnes Ouest I | Tours cedex 9/France | 04/Jun/2019 | 2019T1-11 (2019-000069-19) (CLASSIC MS |
| Ltd. Pineo Medical Ecosystem | Tbilisi/Georgia | 19/Jun/2019 | No reference number used |
| S. Khechinashvili University Clinic Ltd | Tbilisi/Georgia | 17/Jun/2019 | No reference number used |
| Ethik-Kommission der Medizinische Fakultaet der Heinrich Heine Universitaet Duesseldorf-LEC | Dusseldorf/Germany | 20/Dec/2019 | No Reference number, Eudra CT number was stated-CT 2019-000069-19 |
| Ethik-Kommission der Aerztekammer Niedersachsen | Hannover/Germany | 20/Dec/2019 | Bo/39/2019 |
| Ethikkommission an der Universität Regensburg- LEC | Regensburg/Germany | 20/Dec/2019 | 19-1442_1-113 |
| Ethikkommission der Medizinischen Fakultät der Universität Rostock, Institut für Rechtsmedizin-LEC | Rostock/Germany | 20/Dec/2019 | No Reference number, Eudra CT number was stated-CT 2019-000069-19 |
| Segreteria Scientifica Comitato Etico Policlinico di Bari | Bari/Italy | 06/Dec/2019 | studio numero:6042 Prot.n:0102198/06/12/2019-AOUCPG23/COMET/P |
| Azienda Ospedaliero-Universitaria Policlinico-Vittorio Emanuele Catania Comitato Etico | Catania/Italy | 04/Sep/2019 | 0037799/Classifica: TMP/10-2015 |
| Comitato Etico Catania 1 | Catania/Italy | 04/Sep/2019 | 0037799/Classifica: TMP/10-2015 |
| Comitato Etico delle province di Chieti e Pescara | Chieti/Italy | 17/Feb/2020 | 283/2019 |
| Comitato Etico San Martino | Genova/Italy | 11/Jun/2020 | 170/2019 |
| Comitato Etico IRCCS Ospedale S. Raffaele di Milano | Milan/Italy | 07/Nov/2019 | 133/2019 |
| Comitato Etico "Universita Federico ll' | Naples/Italy | 07/Jan/2020 | 263/19 |
| Seconda Università degli Studi di Napoli | Naples/Italy | 02/Jul/2020 | 152/2020 |
| Seconda Università degli Studi di Napoli | Naples/Italy | 02/Jul/2019 | 153/2020 |
| COMITATO ETICO A.S.O. SAN LUIGI GONZAGA | Orbassano/Italy | 16/Aug/2019 | 11524 114/2019 |
| Comitato Etico dell'IRCCS Fondazione Istituto Neurologico Casimiro Mondino di Pavia | Pavia/Italy | 15/Nov/2019 | P – 20190048890 Prot. 20190099744 15/11/2019 |
| Comitato Etico dell'Azienda Ospedaliera S. Andrea di Roma | Rome/Italy | 04/Dec/2019 | 252 SA_2019 |
| Comitato Etico Lazio 1 | Rome/Italy | 31/Mar/2020 | 702 |
| Comitato Etico Lazio 3 | Rome/Italy | 20/Jan/2021 | 221/19 |
| Azienda Socio Sanitaria Territoriale Sette Laghi (Presidio Ospedale di Circolo e Fondazione Macchi) | Varese/Italy | 14/Jul/2020 | 96/2019 |
| IRB of National Cancer Center | Goyang-si/Republic of Korea | 27/Aug/2019 | 2019-0313-0002 |
| IRB of Severance Hospital, Yonsei University Health System | Seoul/Republic of Korea | 15/Jul/2019 | 4-2019-0495 |
| Institutional Review Board | Beirut/Lebanon | 26/Feb/2020 | BIO-2019-0192 |
| Local ethics committee of Bellevue Medical Center | Beirut/Lebanon | 10/Sep/2019 | No reference number on approval |
| Lithuanian Bioethics Committee | Vilnius/Lithuania | 07/Aug/2019 | 188710595 |
| REK Vest | Bergen/Norway | 14/Apr/2020 | 6853 |
| KB przy Uniwersytecie Medycznym im. Karola Marcinkowskiego w Poznaniu Collegium Maius | Poznan/Poland | 19/Jun/2019 | 1113/19 |
| CEIC - Comissão de Ética para a Investigacão Clínica | Lisbon/Portugal | 15/Oct/2019 | MAP / MAP / OF / 2019 / 11127 / 20190568 |
| Comisia Naţională de Bioetică a Medicamentului | Bucharest/Romania | 19/Dec/2019 | 7S/4 |
| SAIH of Kemerovo region “Kemerovo City Clinical Hospital n.a. S.V. Belyaev" | Kemerovo/Russian Federation | 20/Aug/2019 | #96 dd 20Aug2019 |
| Regional EC at SEIHPE “Kursk State Medical University” | Kursk/Russian Federation | 16/Sep/2019 | #6 dd 16Sep2019 |
| IEC at SBIH of Moscow "City Clinical Hospital # 24" | Moscow/Russian Federation | 04/Nov/2019 | #02-11, date 04 Nov 2019 |
| LEC at NIH "Central Clinical Hospital # 2 n.a.N.A. Semashko of OJSC "Russian Railways" | Moscow/Russian Federation | 21/Aug/2019 | #07-19 dd 21Aug2019 |
| LEC at SI “Moscow Regional SRCI n.a. M.F. Vladimirsky” | Moscow/Russian Federation | 12/Sep/2019 | #11 dd 12Sep2019 |
| The Independent Interdisciplinary Ethics Committee on Ethical Review for Clinical Studies | Moscow/Russian Federation | 06/Jun/2019 | #08 dd 17May2019, extract was provided on 06Jun2019 |
| LEC at LLC "MEDIS" | Nizhny Novgorod/Russian Federation | 30/Sep/2019 | #3 dd 30Sep2019 |
| LEC at SBHI of Novosibirsk Region “SNR Clinical Hospital” | Novosibirsk/Russian Federation | 19/Sep/2019 | #3 dd 19Sep2019 |
| SEIHPE "Rostov SMU of MoH of RF" | Rostov-on-Don/Russian Federation | 03/Oct/2019 | 15/19 dd 03Oct2019 |
| EC at City Multifield Hospital #2 | Saint-Petersburg/Russian Federation | 09/Sep/2019 | #8, 09 Sept 2019 |
| LEC at OOO “International Clinic MEDEM” | Saint-Petersburg/Russian Federation | 24/Jul/2019 | #3-07, date 24 Jul 2019 |
| LEC at SBIH "Leningrad Regional Clinical Hospital" | Saint-Petersburg/Russian Federation | 28/Aug/2019 | #8 dd 28Aug2019 |
| Pavlov First Saint Petersburg State Medical University | Saint-Petersburg/Russian Federation | 09/Sep/2019 | #221, date 09 Sept 2019 |
| LEC at SBEI HPE "Samara State Medical University" of the MoH of the RF | Samara/Russian Federation | 31/Jul/2019 | №183 dd 31Jul2019 |
| EC at SBEI HPE "Saratov State Medical University n.a. V. I. Razumovskiy" of the MoH of the RF | Saratov/Russian Federation | 03/Sep/2019 | #1 dd 03Sep2019 |
| LEC at SBEI HPE "Smolensk State Medical University" of the MoH of the RF | Smolensk/Russian Federation | 31/Oct/2019 | №221 dd 31Oct2019 |
| LEC under Siberian State Medical University of Roszdrav | Tomsk/Russian Federation | 30/Sep/2019 | #7787, registration date 16 Sept 2019, meeting date 30 Sept 2019 |
| LEC at SBHI of Yaroslavl Region "Clinical Hospital # 8" | Yaroslavl/Russian Federation | 10/Dec/2019 | #2, date 10 Dec 2019 |
| Ethics Committee of Clinical Center Serbia | Belgrade/Serbia | 27/Jun/2019 | 140/12 |
| Ethics Committee of Clinical Center Nis | Nis/Serbia | 03/Sep/2019 | 31445/3 |
| CEIC Hospital Universitario Ntra Sra de Candelaria | Santa Cruz de Tenerife/Spain | 17/Jul/2019 | 1-4E2K9JU |
| Etikprövningsmyndigheten | Uppsala/Sweden | 14/Aug/2019 | 2019-03886 |
| Commission cantonale d’éthique de la recherche sur l’etre humain(CER-VD) | Lausanne/Switzerland | 10/Dec/2019 | 2019-01720/ 10Dec2019 |
| Comite de protection des personnes Sud | Sfax/Tunisia | 28/Sep/2019 | 0177/2019 / 28Sep2019 |
| Comité local d'éthique de l’Hôpital Militaire Principal d'Instruction de Tunis (HMPIT) | Sfax/Tunisia | 10/Jan/2020 | n/a / 10 Jan 2020 |
| LEC State Institution Institute of Neurology, Psychiatry and Narcology of NAMSU | Kharkiv/Ukraine | 24/Jun/2019 | #6-a date of meeting 21 June 2019, aproval issued 24 June 2019 |
| LEC CI Vinnytsya Regional Psychoneurological Hospital named after O.Yuschenko | Vinnytsia/Ukraine | 13/Jun/2019 | #12.19 date 13 June 2019 |
| NRES Committee Yorkshire & The Humber-South Yorkshire | Leeds/United Kingdom | 30/Sep/2019 | 19/YH/0277 |
| University of Maryland IRB | Baltimore/United States | 25/Jul/2019 | HP-00085222 |
| Copernicus Group IRB | Cary/United States | 31/May/2019 | 20191035 |
| Copernicus Group IRB | Cary/United States | 07/May/2019 | 20191035 |
| Rush University Medical Center | Chicago/United States | 18/Jun/2020 | FWA#00000482 (ORA number: 19060306-IRB02) |
| Oklahoma Medical Research Foundation IRB | Oklahoma City/United States | 11/Jun/2019 | 19-19 |
